# Spontaneously Diabetic Torii (SDT) Fatty Rat, a Novel Animal Model of Type 2 Diabetes Mellitus, Shows Blunted Circadian Rhythms and Melatonin Secretion

**DOI:** 10.1155/2018/9065690

**Published:** 2018-09-23

**Authors:** Katsuya Sakimura, Tatsuya Maekawa, Shin-ichi Kume, Takeshi Ohta

**Affiliations:** ^1^Biological/Pharmacological Research Laboratories, Central Pharmaceutical Research Institute, Japan Tobacco Inc., 1-1 Murasaki-cho, Takatsuki, Osaka, Japan; ^2^Laboratory of Animal Physiology and Functional Anatomy, Graduate School of Agriculture, Kyoto University, Kitashirakawa Oiwake-cho, Sakyo-ku, Kyoto, Japan

## Abstract

In patients with diabetes mellitus (DM), impairments of circadian rhythms, including the sleep–wake cycle, blood pressure, and plasma melatonin concentrations, are frequently observed. Animal models of DM are also reported to show aberrant circadian rhythms. However, the changes in the circadian rhythms of plasma soluble substances, including melatonin, in diabetic animals are controversial. In the present study, we investigated the circadian rhythms of spontaneous locomotor activity, metabolic parameters (plasma glucose, triglyceride, and total cholesterol), and plasma melatonin concentrations in Spontaneously Diabetic Torii (SDT) fatty rats, a novel animal model of type 2 DM. Although SDT fatty rats exhibited low locomotor activity in the dark phase, no phase shifts were observed. The circadian variations of plasma metabolic parameters were more apparent in the SDT fatty rats compared with control Sprague–Dawley (SD) rats. The circadian rhythms of plasma melatonin concentrations were significantly impaired in SDT fatty rats. To get an insight into the mechanism underlying the impaired melatonin secretion in SDT fatty rats, the expression of arylalkylamine N-acetyltransferase (*Aanat*) and acetylserotonin O-methyltransferase (*Asmt*) mRNA, which encode the rate-limiting enzymes for melatonin synthesis, was investigated in the pineal gland. There were no significant differences in *Aanat* and *Asmt* expression between the control SD and SDT fatty rats. These results suggest that SDT fatty rats show impaired circadian rhythms and dysregulated melatonin secretion.

## 1. Introduction

It is well known that mammals have circadian rhythms not only for many kinds of behaviors, such as the sleep–wake cycle and feeding behavior, but also for several fundamental physiological functions, including hormone secretion. The circadian rhythm is organized by the so-called clock genes (e.g., period (*PER*), circadian locomotor output cycles kaput (*CLOCK*), and brain and muscle Arnt-like protein (*BMAL*)) in the suprachiasmatic nucleus (SCN) of the brain as “master regulators,” with the amplitude and/or phase of the rhythm modulated in response to extracellular stimuli such as light [1], feeding [2], and melatonin [3].

Circadian rhythms and metabolic function are known to be reciprocally linked [4]. For example, it has been reported that impairment of circadian rhythms leads to the development of diabetes via the induction of abnormal insulin secretion, decreased sensitivity to insulin, and exacerbated inflammation [5, 6]. In contrast, patients with diabetes reportedly exhibit circadian rhythms of blood pressure and plasma cortisol concentrations [7, 8], suggesting that metabolic functions substantially affect circadian rhythms. However, the mechanisms underlying the disrupted circadian rhythms in diabetes remain to be fully elucidated, because the metabolic disorders are very complex, with many factors, including hyperglycemia, obesity, and hypertension, involved. Several animal models of diabetes mellitus (DM) have been reported to exhibit deficits in circadian rhythms. For example, streptozotocin- (STZ-) induced diabetic rats exhibit a blunted circadian rhythm of locomotor activity with a normal phase entrainment [9], whereas Zucker obese rats, an animal model of type 2 DM (T2DM), exhibit a phase advance and blunted amplitude of locomotor activity [10].

Melatonin, a hormone secreted from the pineal gland in the brain, is known as a regulator of many physiological circadian rhythms including the sleep–wake cycle [11]. Growing evidence suggests an important role for melatonin in DM. For example, melatonin is known to decrease plasma insulin concentrations in humans [12] and rodents [13–15]. Moreover, the physiological increase in nocturnal plasma melatonin concentrations is not observed in diabetic patients, especially those with neuropathy [16]. Similarly, Peschke et al. [17] reported reduced diurnal circulating melatonin levels in patients with T2DM, suggesting that melatonin secretion is impaired in DM patients. Similarly, several animal models of DM have been reported to exhibit aberrant plasma melatonin concentrations. For example, plasma melatonin concentrations at midnight are attenuated in Goto-Kakizaki (GK) rats, and melatonin synthesis efficiency is decreased [18]. Conversely, Peschke et al. [19] reported that melatonin synthesis was increased in the pineal glands of STZ-induced diabetic rats. Moreover, another study demonstrated that diabetic rats transgenic for human islet amyloid polypeptide (HIP rats), an established nonobese model of T2DM, showed normal circadian rhythms and melatonin secretion [20]. Thus, the relationship between metabolic disorders, including hyperglycemia, and aberrant circadian rhythms of melatonin is still controversial.

The Spontaneously Diabetic Torii (SDT) fatty rat is a novel animal model of T2DM, developing not only hyperglycemia but also hyperlipidemia and insulin resistance from a young age [21]. In the present study, we investigated whether SDT fatty rats show impaired circadian rhythms of spontaneous locomotor activity (SLA), plasma metabolic parameters, and plasma melatonin concentrations, as well as changes in the expression of arylalkylamine-N-acetyltransferase (*Aanat*) and acetylserotonin O-methyltransferase (*Asmt*) mRNA, which encode the rate-limiting enzymes for melatonin synthesis [22–24] in the pineal glands of SDT fatty rats.

## 2. Materials and Methods

### 2.1. Animals

The present study was performed in compliance with the Guidelines for Animal Experimentation of Japan Tobacco Biological/Pharmacological Research Laboratories. The animal protocol was designed to minimize pain or discomfort to the animals. SDT fatty rats and age-matched Sprague–Dawley (SD) rats (as controls) were used in the study. The age of animals used in each experiment were as follows: 8 weeks of age for measurement of SLA, plasma glucose, triglyceride (TG), total cholesterol (TC), and melatonin concentrations and 12 weeks of age for measurement of mRNA expressions. All rats were obtained from CLEA Japan (Tokyo, Japan). Rats were housed in groups of two to three per bracket cage in a climate-controlled room (temperature 23°C ± 3°C, humidity 55% ± 15%) under a 12 h light–dark cycle (lights on at 0800 hours), with free access to a commercial diet (CRF-1; Charles River Japan, Yokohama, Japan) and water.

### 2.2. Locomotor Activity

The SLA of rats was assessed using a Supermex apparatus (Muromachi Kikai, Tokyo, Japan). An infrared beam sensor was set on top of a Plexiglas cage, and the number of movements was counted. Activity was integrated every 1 h. SLA was measured during the 12 h light–dark cycle for 5 days. Rats had free access to food and water during the measurements.

### 2.3. Blood Sampling and Measurement of Metabolic Parameters and Melatonin

Blood samples were collected from the tail vein at 1000, 1600, 2100, and 0400 hours by cutting the edge of the tail with a razor. Plasma was separated by centrifugation (15,000 ×g for 5 min at 4°C) and stored at −80°C until analysis. Metabolic parameters, namely, plasma glucose, TG, and TC concentrations, were measured using an automatic analyzer (Hitachi Clinical Analyzer 7180; Hitachi, Tokyo, Japan). Plasma melatonin concentrations were determined using a commercially available ELISA kit (RE54021; IBL International, Hamburg, Germany) according to the manufacturer's instructions.

### 2.4. Sample Preparation for mRNA Measurement

Rats were killed by decapitation, and the pineal gland was dissected at 0400 hours under dim red light. RNA from the pineal gland was extracted using the RNeasy mini kit (Qiagen, Valencia, CA, USA). RNA was quantified using the Nanodrop D8000 (Thermo Scientific, Wilmington, DE, USA). The purity of RNA samples was assessed using the ratio of absorbance at 260/280 nm, and samples with an absorbance ratio of 1.8–2.0 were used to prepare cDNA. The RNA was transcribed into cDNA using high-capacity cDNA reverse transcription kits with RNA inhibitors (Applied Biosystems). The reaction mixture was incubated for 10 min at 25°C, for 2 h at 37°C and then for 5 s at 85°C. The cDNA was stored at −20°C until use.

### 2.5. Quantitative Reverse Transcription Polymerase Chain Reaction

Quantitative reverse transcription polymerase chain reaction (qRT-PCR) was performed in a 20 *μ*L reaction mixture with an automated sequence detector combined with StepOne plus (Applied Biosystems). The reaction mixture was created using TaqMan Gene Expression Master Mix (Applied Biosystems) and contained approximately 50 ng synthesized cDNA, 0.9 *μ*mol/L primers, 0.25 *μ*mol/L probes or TaqMan gene expression assays on demand, and Universal Master Mix (primer/probe set). The reaction mixture was incubated for 2 min at 50°C and then 10 min at 95°C, followed by 40 cycles of 15 s at 95°C and 60 s at 60°C. The expression of beta-actin (*Actb*; purchased from Applied Biosystems), *Aanat* (Rn00664873_g1), and *Asmt* (Rn00595341_m1) was investigated using TaqMan gene expression assays and the Universal Master Mix (primer/probe set), with the expression of *Aanat* and *Asmt* in each sample normalized against that of *Actb*.

### 2.6. Statistical Analysis

Results are expressed as mean ± SEM (standard error of the mean). Comparisons between control SD and SDT fatty rats were performed using two-way analysis of variance (ANOVA) with post hoc tests where appropriate for SLA and plasma parameters over time, whereas qRT-PCR data were compared using unpaired *t*-tests. All data were analyzed using Excel (Microsoft Corp., Bellevue, WA, USA) or EXSUS software (CAC Croit, Tokyo, Japan). A two-sided *P* value of <0.05 was considered significant. A *P* value of <0.05 was considered statistically significant.

## 3. Results

### 3.1. Circadian Rhythms of SLA in SDT Fatty Rats

**Figure 1:**
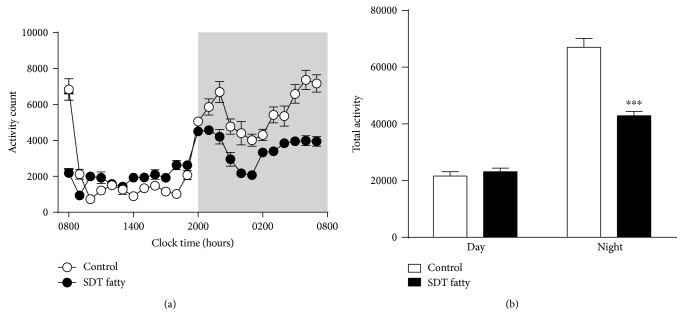
Circadian rhythm of spontaneous locomotor activity (SLA) in control Sprague–Dawley (SD) and Spontaneously Diabetic Torii (SDT) fatty rats. (a) Hourly averages of SLA over a 24 h period for 5 consecutive days under a standard light–dark cycle (the shaded area indicates the dark phase). (b) Total activity during the light and dark phases in control SD and SDT fatty rats. Data are mean ± SEM (*n* = 8 in each group). ^∗∗∗^*P* < 0.001 compared with control SD rats (Aspin-Welch test).

In order to investigate whether there were any disruptions to circadian rhythms of SDT fatty rats, the SLA of control SD and SDT fatty rats was monitored over a period of 5 consecutive days. As shown in Figure 1, SLA during the dark phase was significantly lower in SDT fatty than in control SD rats (*P* < 0.001). In addition, SLA during the first 1 h during the light phase (0800–0900 hours) was markedly decreased in SDT fatty compared with control SD rats. However, the SLA of SDT fatty rats in the light phase was slightly high compared with that of control SD rats, though total activity did not differ significantly between the two groups.

### 3.2. Circadian Rhythms of Metabolic Parameters in SDT Fatty Rats

**Figure 2:**
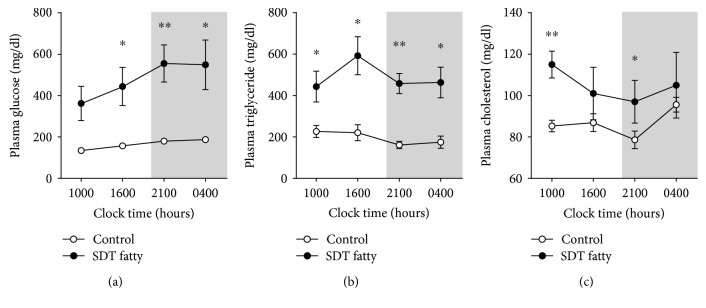
Circadian rhythms of plasma metabolic parameters in control Sprague–Dawley (SD) and Spontaneously Diabetic Torii (SDT) fatty rats: (a) glucose, (b) triglyceride, and (c) total cholesterol. Data are mean ± SEM (*n* = 5 in each group). ^∗^*P* < 0.05 and ^∗∗^*P* < 0.01 compared with the control group at the same time point (two-way ANOVA with post hoc Aspin-Welch tests).

It is well known that several metabolic parameters, including glucose and TG, show circadian rhythms regulated by diet and/or hormones. This led us to investigate whether glucose, TG, and TC exhibit aberrant circadian rhythms in SDT fatty rats. Comparisons of plasma glucose, TG, and TC concentrations in control SD and SDT fatty rats are shown in Figure 2. Two-way ANOVA of the circadian pattern of plasma glucose revealed significant effects of group (*F*_1,8_ = 16.0, *P* < 0.01), time (*F*_3,24_ = 29.3, *P* < 0.001), and their interaction (*F*_3,24_ = 14.4, *P* < 0.001). Post hoc analysis using the Aspin-Welch test revealed that plasma glucose concentrations were significantly higher in SDT fatty than in control SD rats at 1600 (*P* < 0.05), 2100 (*P* < 0.01), and 0400 hours (*P* < 0.05). Two-way ANOVA of the circadian pattern of plasma TG showed significant effects of group (*F*_1,8_ = 17.0, *P* < 0.05) and time (*F*_3,24_ = 4.6, *P* < 0.05). Post hoc analysis using the Aspin-Welch test revealed that plasma TG concentrations were significantly higher in SDT fatty than in control SD rats at all time points measured (i.e., 1000, 1600, and 0400 hours (*P* < 0.05 for all), as well as at 2100 hours (*P* < 0.01)). Unlike glucose and TG concentrations, there were no obvious changes in TC in either strain, with TC concentrations in the range 80–100 mg/dL in control SD rats and 100–120 mg/dL in SDT fatty rats. Plasma TC levels peaked at 0400 hours in control SD rats, compared with 1000 hours in SDT fatty rats. Two-way ANOVA of the circadian pattern of plasma TC showed significant effects of group (*F*_1,8_ = 8.9, *P* < 0.05), but not time (*F*_3,24_ = 2.7, *P* > 0.05). Post hoc analysis using the Aspin-Welch test revealed that plasma TC concentrations were significantly higher in SDT fatty than in control SD rats at 1000 (*P* < 0.01) and 2100 (*P* < 0.05) hours.

### 3.3. Circadian Rhythms of Plasma Melatonin

**Figure 3:**
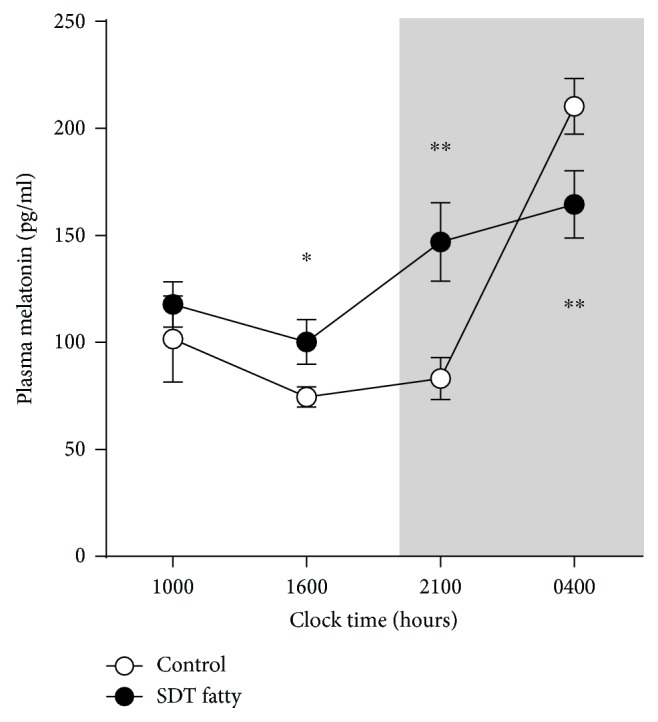
Circadian rhythm of plasma melatonin concentrations in control Sprague–Dawley (SD) and Spontaneously Diabetic Torii (SDT) fatty rats. Data are mean ± SEM (*n* = 5 in each group). ^∗^*P* < 0.05 and ^∗∗^*P* < 0.01 compared with the control group at the same time point (two-way ANOVA with post hoc Aspin-Welch tests).

In order to obtain an insight into the mechanism(s) underlying the aberrant circadian rhythms in SDT fatty rats, we measured plasma melatonin concentrations. As shown in Figure 3, there was an obvious circadian rhythm for melatonin in control SD rats, with low concentrations during the light phase (1000 and 1600 hours) and early dark phase (2100 hours), with the greatest increase in contrast during the night (0400 hours). Two-way ANOVA and subsequent post hoc analysis revealed significant effects of time (*F*_3,42_ = 16.0, *P* < 0.001), but not group (*F*_1,14_ = 1.9, *P* = 0.19). Their interaction was significant (*F*_3,42_ = 7.4, *P* < 0.001) on changes over time in plasma melatonin concentrations between the control SD and SDT fatty rats. Plasma melatonin concentrations were significantly higher in SDT fatty than in control SD rats at 1600 (*P* < 0.05) and 2100 (*P* < 0.01) hours but were significantly lower at 0400 hours (*P* < 0.01). In addition, the total melatonin production was also analyzed, and it was found that there was a higher tendency in SDT fatty rats than in control SD rats, with the area under the curve value of 1949.1 ± 163.0 pg·day/mL (SD rats) vs. 2422.7 ± 206.2 pg·day/mL (SDT fatty rats) (*P* = 0.093).

### 3.4. Expression of Melatonin-Synthesizing Enzymes

**Figure 4:**
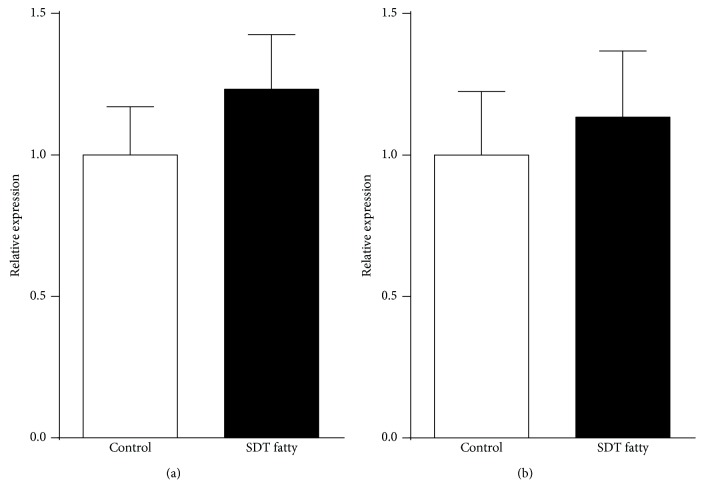
Relative expression of (a) arylalkylamine N-acetyltransferase (*Aanat*) and (b) acetylserotonin O-methyltransferase (*Asmt*) mRNA in the pineal gland of control Sprague–Dawley (SD) and Spontaneously Diabetic Torii (SDT) fatty rats at night (0400 hours). Data are mean + SEM (*n* = 6 in each group). There were no significant differences in *Aanat* and *Asmt* expression between the two strains (*P* > 0.05, Student's *t*-test).

The observation that SDT fatty rats showed blunted circadian rhythms of plasma melatonin led us to investigate whether the expression of *Aanat* and *Asmt* mRNA, enzymes important for melatonin synthesis, in the pineal gland of SDT fatty rats was impaired compared with that of control SD rats at night, when melatonin expression was highest. Although there were no significant differences in *Aanat* and *Asmt* mRNA expression in the pineal glands of the two groups, there was a tendency for higher *Aanat* and *Asmt* mRNA expression in SDT fatty than in control SD rats (Figure 4).

## 4. Discussion

In the present study, we found that (1) SDT fatty rats showed aberrant circadian rhythms of SLA, (2) the circadian fluctuations of plasma glucose, TG, and TC concentrations were found to be more apparent in SDT fatty rats, and (3) these rats exhibited blunted circadian rhythms of plasma melatonin secretion, even though the mRNA expression of melatonin-synthesizing enzymes did not differ significantly from that in the control SD rats.

Because rats are generally nocturnal, their activity increases in the dark phase and decreases markedly during the light phase. However, SLA in SDT fatty rats was decreased during the dark phase, with a tendency for higher SLA compared with control SD rats during the light phase. This observation suggests that SDT fatty rats have blunted circadian rhythms of SLA. In addition, the entrainment by light stimuli seemed normal in the SDT fatty rats, as revealed by the observation that the SDT fatty rats exhibited the same SLA pattern over a period of 5 consecutive days of monitoring, although actograms were not measured in the present study. The findings of the present study confirm those of previous reports demonstrating impaired circadian rhythms in animal models of T2DM. For example, Zucker obese rats, an animal model for T2DM with mutations in the leptin receptor, exhibit a phase advance and a decreased amplitude of activity and body temperature [10]. In addition, Otsuka Long-Evans Tokushima Fatty (OLETF) rats have been reported to show decreased nocturnal locomotor activity before developing hyperglycemia [25]. Furthermore, STZ-induced diabetic rats, an animal model of type 1 DM, also show a blunted circadian rhythm for locomotor activity, but no phase shift [9]. Together, these findings suggest that hyperglycemia and/or obesity may induce blunted circadian rhythms while phase entrainment is preserved.

Circadian rhythms are known to be affected by food-taking behavior [26, 27]. As our previous studies have revealed that SDT fatty rats show hyperphagia [28, 29], this feature may underlie the deficits of circadian rhythm in the SDT fatty rats. Although there is no data on the food intake pattern of SDT fatty rats, the relatively higher SLA during the light phase may reflect the food-taking behavior. Consistent with this, plasma glucose, TG, and TC concentrations are significantly higher than those of control SD rats even in the light phase as well as in the dark phase.

As mentioned in Introduction, diabetes and impaired circadian rhythms are known to be closely linked [4]. In addition, Yoda et al. [30] recently reported that the increased HbA1c associated with poor glycemic control in T2DM induces sleep disorder with exacerbated sleep quality. Furthermore, those authors demonstrated that sleep disorders in T2DM can increase the risk of cardiovascular events via aberrant hypertension in the morning [30]. Future studies investigating the relationship between hyperglycemia and the quality of sleep in SDT fatty rats are needed in order to elucidate the underlying pathophysiology.

In addition to the impaired circadian rhythm of SLA in SDT fatty rats, the rhythm of melatonin secretion was blunted in SDT fatty compared with control SD rats. Melatonin signaling, including its synthesis and secretion, is known to be suppressed by light inputs from eyes and usually reaches peak values at night both in humans and in rodents [31–33]. Consistent with this, in the present study, melatonin concentrations in control SD rats were low during the light and early dark phases and were highest in samples collected at 0400 hours. In contrast, melatonin concentrations during the light phase (1600 hours) and at 2100 hours were higher in SDT fatty than in control SD rats, whereas they were lower at 0400 hours in the SDT fatty rats, suggesting that SDT fatty rats have blunted circadian rhythms of melatonin secretion. To get an insight into the mechanism underlying this aberrant melatonin secretion, we investigated the mRNA expression of melatonin-synthesizing enzymes in the pineal glands of SDT fatty rats by focusing on the peak time of melatonin secretion (0400 hours). Unexpectedly, there was no significant difference in the expression of *Aanat* and *Asmt* between the SDT fatty and control SD rats, while there was a tendency for higher expression in the SDT fatty rats. In the present study, we did not investigate the levels of Aanat and Asmt proteins, nor the precursors of melatonin including tryptophan and serotonin in the pineal gland of SDT fatty rats. Thus, further study is required in order to expand the understanding about the alteration of melatonin synthesis of SDT fatty rats. One possibility is that the not obvious changes in the mRNA expression in the SDT fatty rats were due to a time lag between the mRNA expression and the protein production. Thus, in addition to the time course measurement of melatonin protein, the time course of mRNA expression is needed in order to unveil the exact melatonin synthesis regulation in the SDT fatty rats. Similar findings have been reported by Frese et al. [18] who demonstrated that melatonin synthesis was impaired in the pineal glands of GK rats. They also found that the mRNA expression of three of four melatonin-synthesizing enzymes (i.e., tryptophan hydroxylase, aromatic amino acid decarboxylase, and A*smt*) was significantly increased in the pineal gland of GK rats [18]. Based on these findings, the authors suggested that the upregulated mRNA expression may be a compensatory response to a decrease in the efficiency of melatonin synthesis. Although melatonin content and its synthesis in the pineal gland of SDT fatty rats were not investigated in the present study, it may be that a similar compensatory response occurs in SDT fatty rats. Meanwhile, the mRNA expression of *Aanat* was decreased by approximately 50% in GK rats in contrast to SDT fatty rats [18]. The findings from the present study confirm the findings of Frese et al. [18] in part; however, the opposite direction of changes in *Aanat* mRNA expression between the two studies suggests that the mechanism regulating melatonin synthesis in the pineal gland may differ between these two rat strains.

Altered melatonin secretion in diabetic patients has also been reported. For example, the physiological increase in nocturnal plasma melatonin concentrations was not observed in diabetic patients, especially those with neuropathy [16]. Similarly, Peschke et al. [17] reported reduced diurnal circulating melatonin levels in T2DM patients. The findings of the present study support these clinical findings. Moreover, a relatively large clinical case-control study suggested that the decreased nocturnal melatonin secretion may be the cause of T2DM [34]. It is important to investigate any intervention such as antidiabetics that will improve the circadian rhythm deficits as well as the symptoms of T2DM. Such an investigation should be performed in the future in order to judge the validity of this model as T2DM is comorbid with circadian rhythm deficit.

## 5. Conclusions

The results of the present study provide important evidence of the deficits in the circadian rhythms, as well as dysregulation of melatonin secretion, in animal models of T2DM. Unlike other animal models of DM, SDT fatty rats develop not only hyperglycemia but also hyperlipidemia and insulin resistance from a young age as described in Introduction. Thus, given the findings in this study, SDT fatty rats may be a useful animal model for investigating the relationship between deficits in the circadian rhythm and metabolic dysfunction.

## Data Availability

The data used to support the findings of this study are available from the corresponding author upon request.
